# Comparison of minimal residual disease detection in multiple myeloma between the DuraClone and EuroFlow methods

**DOI:** 10.1038/s41598-021-89761-9

**Published:** 2021-05-27

**Authors:** Takeshi Yoroidaka, Kentaro Narita, Hiroyuki Takamatsu, Momoko Fujisawa, Shinji Nakao, Kosei Matsue

**Affiliations:** 1 Department of Hematology, Faculty of Medicine, Institute of Medical, Pharmaceutical and Health Sciences, Kanazawa University, 13-1 Takaramachi, Kanazawa, Ishikawa 920-8641 Japan; 2 Division of Hematology/Oncology, Department of Medicine, Kameda Medical Center, 929 Higashi-chou, Kamogawa-shi, Chiba, 296-8602 Japan

**Keywords:** Myeloma, Prognostic markers

## Abstract

In this study, the minimal residual disease (MRD) levels in patients with multiple myeloma (MM) were assessed by comparing the new 8-color single-tube multiparameter flow cytometry method (DuraClone), which reduces the cost of antibodies and labor burden of laboratories, with the EuroFlow next-generation flow (NGF) method. A total of 96 samples derived from 69 patients with MM were assessed to determine the total cell acquisition number (tCAN), percentages of total and normal plasma cells (PCs), and MRD levels using two methods. We found that the tCAN was significantly higher with EuroFlow-NGF than with DuraClone (median 8.6 × 10^6^ vs. 5.7 × 10^6^; *p* < 0.0001). In addition, a significant correlation in the MRD levels between the two methods was noted (r = 0.92, *p* < 0.0001). However, in the qualitative analysis, 5.2% (5/96) of the samples showed discrepancies in the MRD levels. In conclusion, the DuraClone is a good option to evaluate MRD in multiple myeloma but it should be used with caution.

## Introduction

Recently, an increasing number of patients with multiple myeloma (MM) achieve complete response (CR) because of novel agents, and this is well associated with good progression-free survival and overall survival^1–4^. The minimal residual disease (MRD) levels are used to stratify patients who achieve CR and predict their outcomes^5–9^. In 2016, the International Myeloma Working Group (IMWG) approved the definition of the MRD criteria, such as Flow-MRD negative and Sequencing MRD negative^10^. A Flow-MRD-negative case is assessed by multiparameter flow cytometry (MFC) with a minimum sensitivity of 1/10^5^ nucleated cells and equivalent methods, such as EuroFlow next-generation flow (NGF). EuroFlow-NGF, which uses two 8-color tubes, is a highly sensitive (cutoff value, 2 × 10^−6^) and standardized way of detecting MRD^11^, and the IMWG advocates the use of EuroFlow or equivalent methods to assess MRD. Although it is more rapid and cost-effective in detecting MRD compared with next-generation sequencing^5,6,9,12,13^, the EuroFlow method is too expensive (< 350 USD/sample) to perform under public medical insurances in some countries, including Japan^12,14^. The DuraClone RE PC kit (DuraClone panel) is a recently developed 8-color single-tube dry antibody panel that uses the Kaluza software (Beckman Coulter, Brea, USA) for the identification of abnormal plasma cells (aPCs) to detect MRD in MM with a sensitivity of 1.0 × 10^−5^^15^. Although the DuraClone panel is widely available, whether it can reduce the cost of antibodies and labor burden of laboratories remains to be validated, and the relationships in the PCs and MRD detection between EuroFlow-NGF and DuraClone are unclear. Therefore, we hypothesized that the DuraClone method could offer the same performance as that offered by EuroFlow-NGF for the detection of MRD in patients with MM. In this study, we performed a comparison between the two methods.

## Methods

### Patients and samples

The MRD test was prospectively performed in patients with IMWG-defined symptomatic MM who were treated at Kameda Medical Center and Kanazawa University Hospital in Japan from January 2017 to November 2018. As part of routine clinical care, 4 mL of bone marrow aspirate (first bone marrow aspiration pull) was collected in tubes containing ethylenediaminetetraacetic acid. The samples were then mixed, measured, and split evenly (2 mL each). One sample was analyzed with the DuraClone 8-color single-tube panel at Kameda Medical Center, and the other was analyzed with the EuroFlow-NGF 8-color 2-tube panel at Kanazawa University Hospital. Within 48 h after collection, up to 20 million cells or the entire volume of the sample was used. All study methods were carried out in accordance with the Declaration of Helsinki.

### Eight-color MFC analysis using the DuraClone and EuroFlow-NGF methods

The technical details and validation of the EuroFlow-NGF have been reported previously^11^. In brief, the EuroFlow method uses ammonium chloride-based bulk lysis, followed by surface staining using antibodies against CD138-BV421, CD27-BV510, CD38 multiepitope (ME)-FITC, CD56-PE, CD45-PerCP Cy5.5, CD19-PECy7, CD117-APC, and CD81-APC-C750 in Tube 1 and surface/intracellular staining using antibodies against CD138-BV421, CD27-BV510, CD38 ME-FITC, CD56-PE, CD45-PerCP Cy5.5, CD19-PECy7, cytoplasmic (cy) Igκ-APC, and cyIgλ-APC-C750 after permeabilization in Tube 2, as previously described^11,14^. Anti-CD38ME antibody was used to prevent the interference of anti-CD38 monoclonal antibody such as daratumumab. The FACSCanto II (BD Biosciences) flow cytometer was used to acquire all samples, and the gating and identification of clonal aPCs were manually performed by experts using the Infinicyt software (Cytognos, Salamanca, Spain) for the EuroFlow-NGF method.

**Figure 1:**
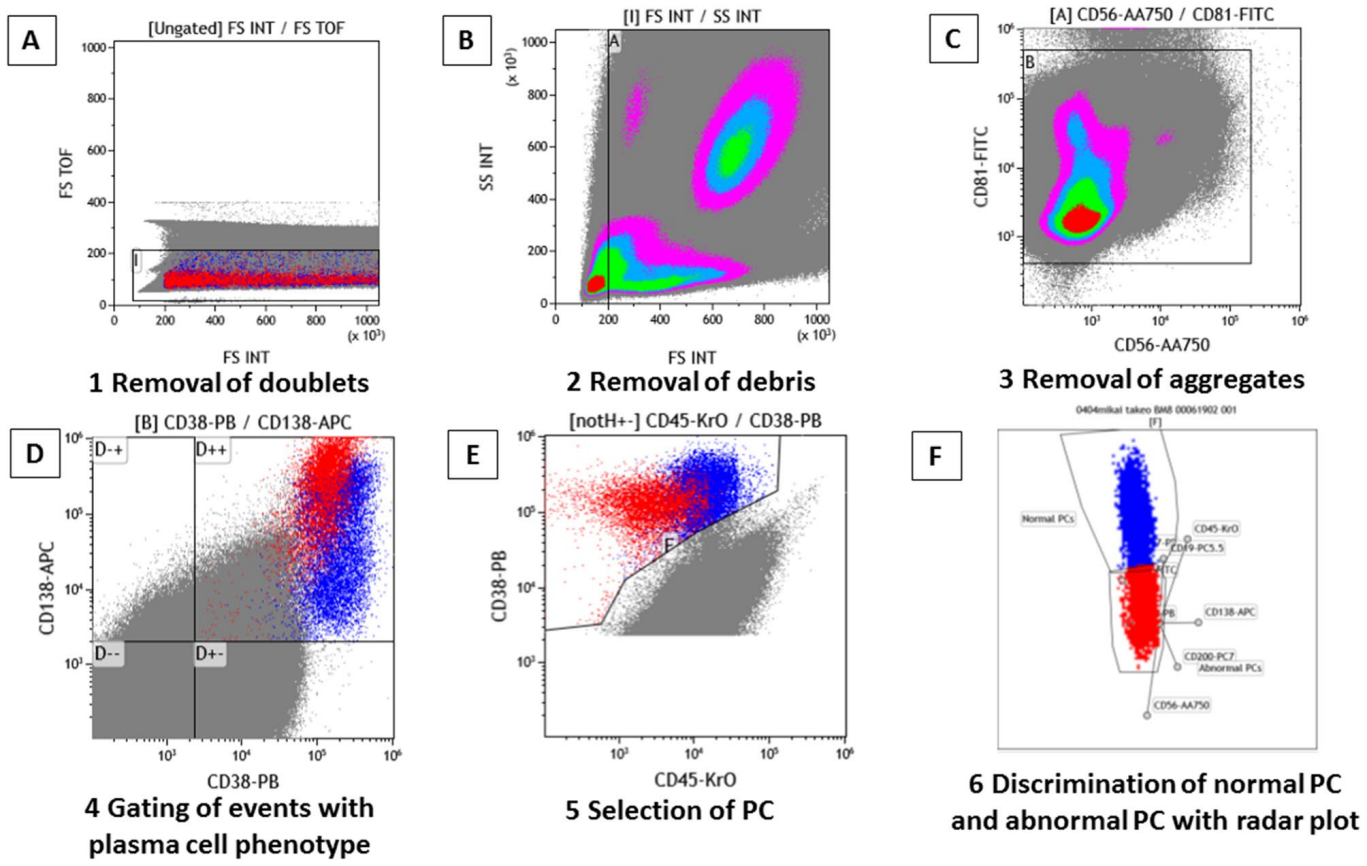
Overview of the gating strategy of plasma cells (PCs) and discrimination of normal and abnormal PCs with radar plot by DuraClone method in patients who did not receive anti-CD38 antibody. Light scatter characteristics are used to exclude doublets with high time of flight (TOF) (**A**), debris with lower scatter peak than lymphocytes (**B**) and aggregates with high fluorescent events with CD81-FITC and CD56-APC-A750 (**C**). All possible plasma cells are captured by gating CD38 + and CD138 + bright events (**D**). PCs are selected with CD38 high events using CD45 (**E**). Abnormal PCs are detected with radar plot (**F**). Red dots: myeloma cells, Blue dots: normal plasma cells. *FS TOF* forward scatter time of flight, *FS INT* forward scatter intensity, *SS INT* side scatter intensity.

**Figure 2:**
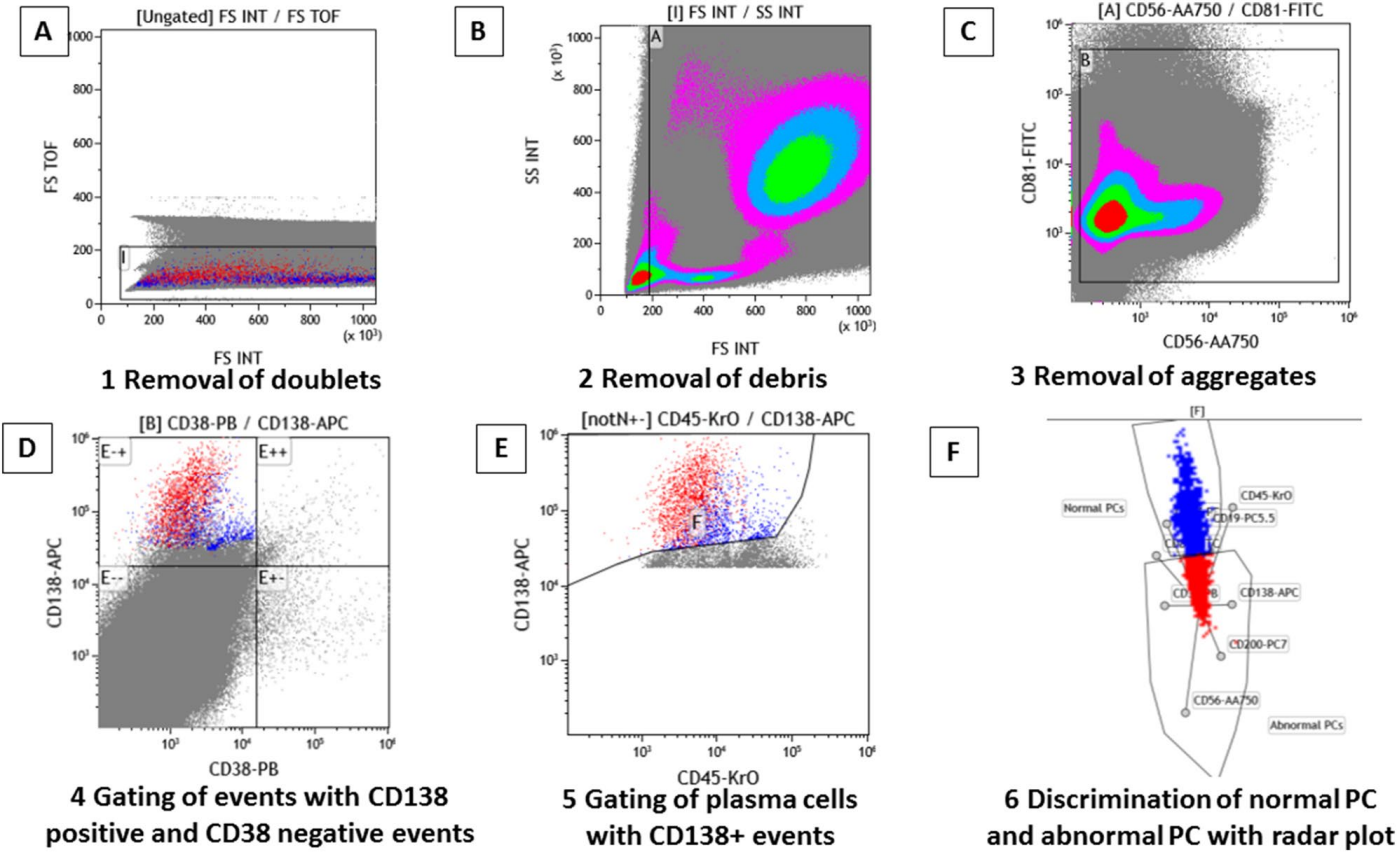
Overview of the gating strategy of plasma cells and discrimination of normal and abnormal plasma cells with radar plot by DuraClone method in patients who received anti-CD38 antibody. Light scatter characteristics are used to exclude doublets with high time of flight (TOF) (**A**), debris with lower scatter peak than lymphocytes (**B**) and aggregates with high fluorescent events with CD81-FITC and CD56-APC-A750 (**C**). PCs are captured by gating CD138 + CD38 − events (**D**). PCs are selected with CD138 high events using CD45 (**E**) Abnormal PCs are detected with radar plot (**F**). Red dots: myeloma cells, Blue dots: normal plasma cells. *FS TOF* forward scatter time of flight, *FS INT* forward scatter intensity, *SS INT* side scatter intensity.

**Figure 3:**
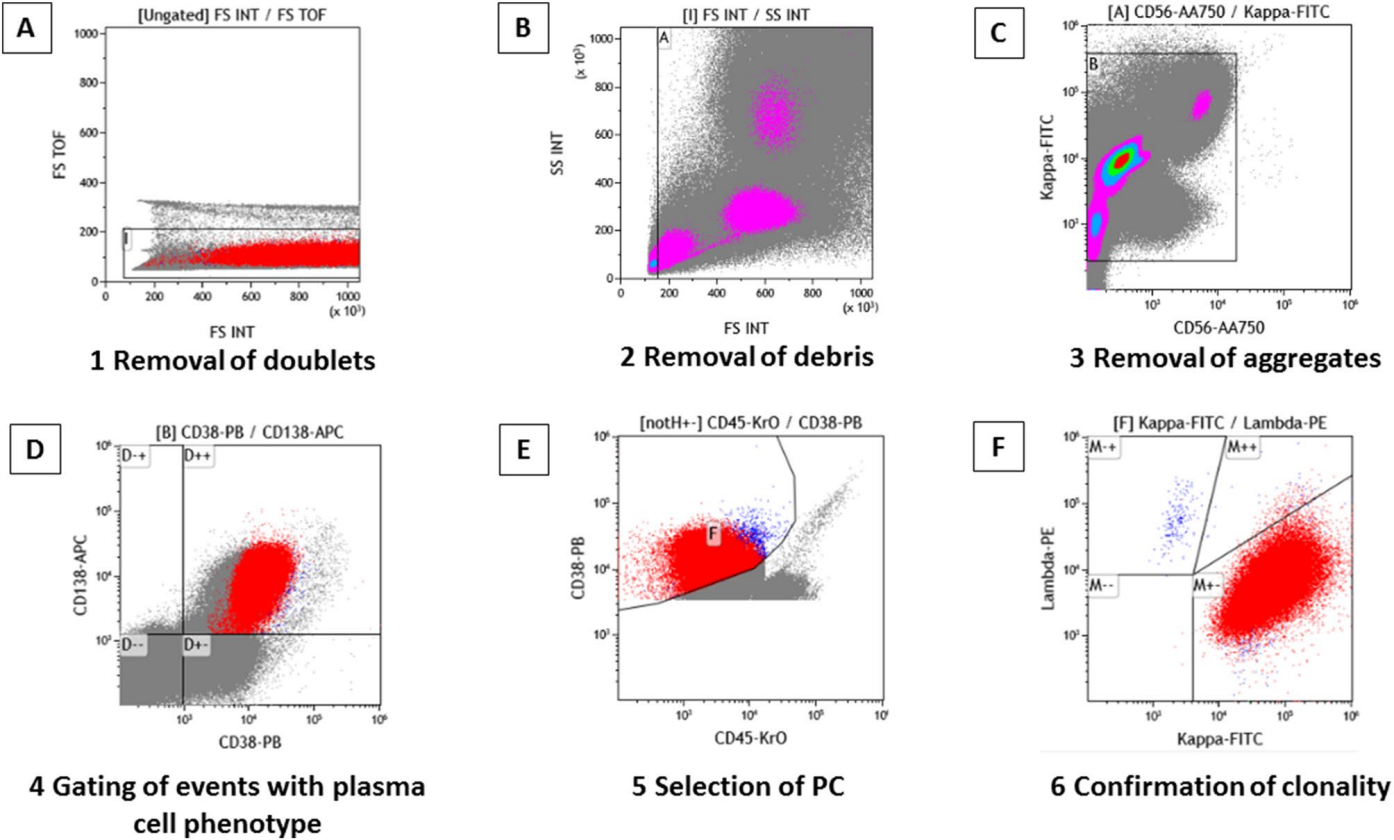
Overview of the gating strategy of plasma cells (PCs) and confirmation of clonality of abnormal PCs by DuraClone method. Light scatter characteristics are used to exclude doublets with high time of flight (TOF) (**A**), debris with lower scatter peak than lymphocytes (**B**) and aggregates with high fluorescent events with Ig-κ-FITC and CD56-APC-A750 (**C**). All possible PCs are captured by gating CD38 + and CD138 + bright events (**D**). PCs are selected with CD38 high events using CD45 (**E**). Confirmation of clonality by Igκ and Igλ (**F**).

On the other hand, the DuraClone method^15^ was performed with fixative-free erythrocyte lysis (VersaLyse Lysing Solution, Beckman Coulter), followed by surface staining with the prefixed dry reagent of antibodies against CD138-APC, CD38-PB (non-ME antibody), CD56-APC-A750, CD19-PC5.5, CD45-KrO, CD200-PC7, CD81-FITC, and CD27-PE in a single tube to identify aPCs. The Navios flow cytometer (Beckman Coulter, USA) was used to acquire the samples. Cell doublets were excluded by omitting the events with high time of flight (TOF)^15^ (Fig. 1A) and debris were excluded by omitting the events with lower forward scatter peak signals than lymphocytes (Fig. 1B). Furthermore, dye aggregates were eliminated with excluding the high fluorescent events with CD81-FITC and CD56-APC-A750 (Fig. 1C). Following these procedures, primary identification of plasma cells was performed by gating the events with CD38-PB high and CD138-APC high (Fig. 1D). Additionally, plasma cells were selected with the gating of CD38 and CD45 (Fig. 1E). The samples following anti-CD38 monoclonal antibody treatment, removal of doublets (Fig. 2A), debris (Fig. 2B), and aggregates (Fig. 2C) was performed as well as the samples without using anti-CD38 monoclonal antibody, but primary identification of plasma cells was performed CD38-PB negative and CD138-APC high (Fig. 2D). Additionally, plasma cells are selected with the gating of CD138 and CD45 (Fig. 2E), and identification of aPCs was manually performed using a radar plot, which is a projection of the intensity of the released light energy from each eight fluorochrome on a 2-dimensional series of spokes projecting from a central point analyzed by the Kaluza v1.5a software (Beckman Coulter) as previously described in both samples with or without using anti-CD38 monoclonal antibody^15^ (Figs. 1F, 2F). Using DuraClone, the analysis strategy bases on the different-from-normal approach as there is no specific marker of abnormality known yet but only deviations from the normal expression pattern which are CD45med, CD38high, CD138med/high, CD19positive, CD56dim/neg, CD27high, CD81high, CD200dim/neg. Abnormal plasma cells can deviate by a diversity of abnormal patterns, e.g. CD45low/neg, CD38med, CD138 all high, CD19neg, CD56dim/neg, CD27dim, CD81high, CD200high, or as another example, CD45med, CD38high, CD138med/high, CD19neg, CD56high, CD27med, CD81dim/neg, CD200dim/neg^16^. In addition to original method of surface staining (Tube 1), we additionally performed intracellular staining of cyIgκ-FITC and cyIgλ-PE (DAKO, product code: FR481) concomitant with CD38-PB (non-ME antibody, Beckman Coulter, product code: B09683), CD138-APC (Beckman Coulter, product code: A87787), CD19-PC5.5 (Beckman Coulter, product code: A66328), CD56-APC-A750 (Beckman Coulter, product code; B46024), CD45-KrO (Beckman Coulter, product code: B36294), CD200-PC7 (Beckman Coulter, product code: B43299) after permeabilization with PerFix (Beckman Coulter) in Tube 2 to confirm the clonality of the abnormal cells as our original method. In Tube 2, elimination of cell doublets and debris were performed by omitting the events with high TOF (Fig. 3A) and by omitting the events with lower forward scatter peak signals than lymphocytes (Fig. 3B) as performed in Tube 1. Furthermore, dye aggregates were eliminated with excluding the high fluorescent events with using cyIgκ-FITC and CD56-APC-A750 instead of CD81-FITC and CD56-APC-A750 in Tube 1 (Fig. 3C). Primary identification of plasma cells was performed by gating the events with CD38-PB high and CD138-APC high in samples without the treatment of anti-CD38 antibody (Fig. 3D) and with CD38-PB negative and CD138-APC high in those with the treatment of anti-CD38 antibody. Plasma cells were selected with the gating of CD45-KrO and CD38-PB (Fig. 3E) in samples without the treatment of anti-CD38 antibody or CD45-KrO and CD138-APC in those with the treatment of anti-CD38 antibody, and clonality of plasma cells were confirmed by cyIgκ-FITC and cyIgλ-PE (Fig. 3F). We splitted evenly (1 mL each) for surface staining and intracellular staining in the DuraClone analysis. These two tubes were not merged in DuraClone analysis. The correlation of the total cell acquisition number, percentages of the total and normal PCs, and MRD levels in both methods was then analyzed. In the DuraClone analysis, total cell acquisition number (Tube 1 + Tube 2, 2 mL-BM), percentages of the total and normal PCs, and MRD levels of Tube 1 (1 mL-BM) was adopted for comparison with EuroFlow-NGF. In the MRD level analysis, detection of at least 20 aPCs was required for their precise identification in both methods. When 1 × 10^7^ cells were analyzed using EuroFlow-NGF, the lower limit of detection (LOD) was set at 2.0 × 10^−6^ and LOD of DuraClone was set at 4.0 × 10^−6^. Matching of target channels was verified daily with a new calibration run to prevent target mismatch and all instruments underwent daily verification of optical alignment and fluidics using other calibration bead particles (Flow Check beads, Beckman Coulter). Regarding EuroFlow-NGF, the monitoring of instrument performance was done according to the EuroFlow Standard Operating Protocol (SOP) for Instrument Setup and Compensation (https://www.euroflow.org/protocols).

### Statistical analysis

Wilcoxon signed-rank test was used for the comparison of paired variables. Spearman’s correlation coefficient was applied for evaluating the correlation of paired data. All statistical analyses were performed using the EZR software package (Saitama Medical Center/Jichi Medical University, Saitama, Japan)^17^, which is a graphical user interface for R version 3 0.4.0 (R Foundation for Statistical Computing, Vienna, Austria). *P* values < 0.05 were considered statistically significant (two-sided).

### Ethics

The local ethic committees of Kameda Medical Center and Kanazawa University approved this study.

### Consent to participate

All patients provided written consent in accordance with the Declaration of Helsinki.

### Consent for publication

All patients provided written consent in accordance with the Declaration of Helsinki.

## Results

**Figure 4:**
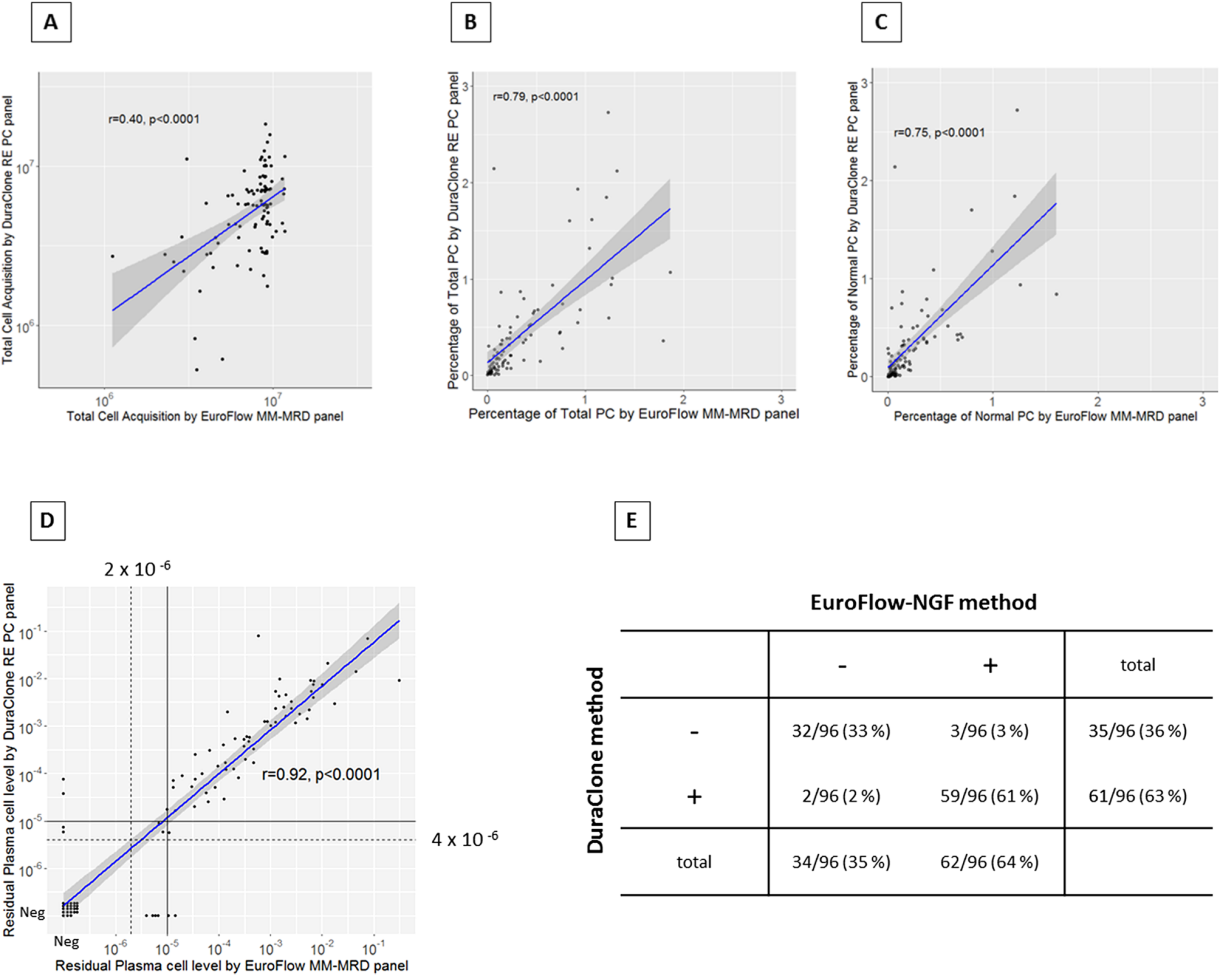
Comparison between the DuraClone and EuroFlow-NGF methods. Total cell acquisition (**A**), percentages of total plasma cells (PCs) (**B**) and normal PCs (**C**), quantitative study of minimal residual disease (MRD) detection (**D**), and qualitative study of MRD detection (**E**).

A total of 96 samples derived from 69 patients were analyzed, and 21 patients were assessed repeatedly in different states of disease. The patient characteristics (diagnosis and clinical status by the IMWG response criteria for MRD evaluation) are summarized in Table 1. The number of cells obtained using EuroFlow-NGF (median 8.6 × 10^6^; range 1.1 × 10^6^–11.7 × 10^6^) was significantly higher than that obtained by the DuraClone panel (median 5.7 × 10^6^; range 0.5 × 10^6^–18.3 × 10^6^; *p* < 0.0001), and the correlation was not so high (r = 0.40; *p* < 0.0001) (Fig. 4A). In the 91 (94.8%) and 75 (78.1%) of 96 samples assessed by the EuroFlow-NGF and DuraClone, respectively, > 3 million cells were acquired as recommended by the NCI myeloma working group panel^18^. In addition, 81 (84.3%) and 56 (58.3%) of the 96 samples assessed by the EuroFlow-NGF and DuraClone methods, respectively, achieved an acquisition of 5 million cells as recommended by the IMWG^10^.

**Table 1:** Characteristics of the patients' diagnosis and clinical status when each sample was analyzed.

Diagnosis (69 patients)		Clinical status (96 samples)	
IgGκ	31 (44.9%)	PR	5 (5.2%)
IgGλ	8 (11.5%)	VGPR	28 (29.1%)
IgAκ	9 (13.0%)	CR	14 (14.5%)
IgAλ	8 (11.5%)	sCR	41 (42.7%)
BJκ	5 (7.2%)	CLR	8 (8.3%)
BJλ	8 (11.5%)		

*CR* complete response, *sCR* stringent CR, *VGPR* very good partial response, *PR* partial response, *CLR* clinical relapse

Next, we compared the percentages of the total and normal PCs between the EuroFlow-NGF and DuraClone methods. Relatively a high-correlation was noted in the percentages of the total (r = 0.79; *p* < 0.0001) and normal (r = 0.75; *p* < 0.0001) PCs (Fig. 4B–C).

Finally, we compared the efficiency of MRD detection between the two methods. In the quantification of the MRD levels, a high-correlation in MRD levels was observed between the two methods (r = 0.92; *p* < 0.0001) (Fig. 4D). Conversely, in the qualitative study of MRD detection, 5 samples (5.2%) showed a disagreement of MRD negativity when a cutoff value of 10^−5^ was applied (Fig. 4E, Table 2). Among these samples, three were found to be MRD positive by EuroFlow-NGF and MRD negative by DuraClone, and the total number of cells acquired by EuroFlow-NGF (median 8.4 × 10^6^, range 7.3 × 10^6^–8.7 × 10^6^) was almost the same with that acquired by the DuraClone panel except for sample No. 5 (median 8.7 × 10^6^ , range 2.2 × 10^6^–10.0 × 10^6^; *p* = 0.7). In contrast, two samples were found to be MRD negative by EuroFlow-NGF and MRD positive by DuraClone, judged as such using the following antigens: 1 sample by CD56 only and 1 sample by CD56 and CD200 (Fig. 4E and Table 2).

**Table 2:** Overview of the samples showing discrepancy of MRD negativity between DuraClone and EuroFlow-NGF methods.

Sample no	Clinical status	Diagnosis	DuraClone						EuroFlow-NGF				PFS, post-MRD assessment (months)
			tCAN	Total cell of tube1	Abnormal PC	Abnormal PC ratio (MRD)	Abberant expression	MRD negativity^a^	tCAN	Abnormal PC	Abnormal PC ratio (MRD)	MRD negativity^a^	
1	CR	IgGK	28,53,031	13,91,147	100	7.1 × 10^−5^	CD56 +	Positive	91,70,213	0	< 2 × 10^−6^	Negative	No progression (48)
2	sCR	IgAK	1,42,04,476	70,82,588	268	3.7 × 10^−5^	CD56 + , CD200 +	Positive	92,29,865	0	< 2 × 10^−6^	Negative	No progression (35)
3	CR	BJK	1,09,44,839	56,55,587	32	5.6 × 10^−6^	–	Negative	84,12,160	94	1.1 × 10^−5^	Positive	NA
4	CR	BJK	87,01,500	44,10,891	6	< 4 × 10^−6^	–	Negative	87,41,417	127	1.5 × 10^−5^	Positive	No progression (12)
5	VGPR	IgGL	22,42,709	10,08,320	6	< 4 × 10^−6^	–	Negative	73,41,906	78	1.1 × 10^−5^	Positive	No progression (29)

*tCAN* total cell acquisition number, *PC* plasma cells, *MRD* minimal residual disease, *NGF* next-generation flow, *CR* complete response, *sCR* stringent CR, *VGPR* very good partial response, *PFS* progressionfree survival, *NA* not assessed due to be transferred to another hospital.

^a^The thresholds of MRD negativity was 1 × 10^−5^ in both DuraClone and EuroFlow-NGF.

## Discussion

This study demonstrated that the DuraClone method was not comparable to EuroFlow-NGF in total cell acquisition number, but comparable in percentage of total/normal PCs, and MRD levels. Although at least 3 million cells and ideally 5 million cells should be analyzed to achieve the lower limit of detection, i.e., 1.0 × 10^−5^ according to the recent guidelines^19^, only 58.3% (56/96) of the samples achieved over 5 million total nucleated cells using DuraClone. On the contrary, 84.3% (81/96) achieved this number using EuroFlow-NGF. This difference likely resulted because the DuraClone method utilized the fixative-free erythrocyte lysis process, which tends to lose the nucleated cells, contrary to the bulk lysis in the EuroFlow-NGF method, as previously reported^11^. In addition to that reason, in our modified DuraClone analysis, we used intracellular staining including cyIgκ and cyIgλ (Tube 2) for the confirmation of clonality, which cannot merge with surface staining (Tube 1) as EuroFlow-NGF can. We compared 1 mL sample of Tube 1 of DuraClone with 2 mL sample of EuroFlow-NGF for percentages of total and normal PCs and MRD, because Tube 1 of DuraClone was designed for detection of MRD, but Tube 2 was our institutional original method for just confirmation of clonality, not for detection of MRD. Confirmation of clonality by using cyIgκ and cyIgλ is helpful for the detection of MRD.

An inconsistent MRD negativity between the EuroFlow-NGF and DuraClone methods was noted in 5 samples (5.2%). We consider that one of reasons of inconsistent MRD negativities of these samples is that these inconsistencies are recognized in the samples with low MRD level and 3/5 of MRD positive samples (all three samples are MRD positive in EuroFlow-NGF) are MRD level of 1–2 × 10^−5^ as shown in Table 2. Regarding the difference of tCAN, because tCAN of sample No.5 by DuraClone was much less than that by EuroFlow-NGF, the inconsistency of MRD could be due to the little tCAN by DuraClone. The difference between the antibody panels, in addition to tCAN, may also have resulted in the inconsistent cases between the EuroFlow-NGF and DuraClone methods. The DuraClone method adopted antibodies against CD200 instead of CD117, cyIgκ, and cyIgλ, which were used in the EuroFlow-NGF panel^11^. CyIgκ and cyIgλ are extremely important to confirm the clonality of myeloma cells, and thus, ruling out the polyclonal cells, which express aberrant antigens such as CD56 and CD200, is impossible without anti-cyIgκ and anti-cyIgλ antibodies. For example, Flores-Montero et al. reported that anti-CD200 antibody demonstrated low contribution as a potential MRD marker because it was heterogeneously expressed in the normal bone marrow PCs from 28/28 samples (median percentage of CD200 + normal PCs, 49%; range 40–68%) as well as in 8/20 (40%) cases with abnormal PC populations (median percentage of CD200 + aPCs, 100%; range 58–100%)^11^, thus using CD200 for detecting aPCs has the potential to overestimate MRD. Recently, Roshal et al. reported that the MSKCC 10-color single-tube and EuroFlow-NGF 8-color 2-tube methods exhibited similar efficiency in assessing MRD^20^; in both methods, anti-cyIgκ and anti-cyIgλ antibodies were adopted to confirm the clonality of aPCs. In our analysis, 2 of 5 samples were MRD positive by DuraClone method and negative by EuroFlow-NGF (samples No. 1 and 2 in Table 2). CD19 negative/CD56 positive cells in sample No. 1 and CD200 positive/CD19 negative/CD56 positive cells in sample No. 2 were detected as aPCs by DuraClone analysis. In both samples, we performed cyIgκ and cyIgλ analysis for confirmation of clonality in Tube 2 of DuraClone, but no clonality was observed. In addition to the reason that CD200 would overestimate aPCs in Tube 1, we hypothesized that the reason of difference of MRD might be owing to the difference of approach for detecting aPCs between both methods. EuroFlow-NGF used cyIgκ and cyIgλ for counting aPCs in addition to surface staining by the fusion of both surface and cytoplasmic staining tubes, however, DuraClone could not fuse surface and cytoplasmic staining cells together, thus counting of aPCs was performed only by surface staining tube using the radar plot. For these reasons, DuraClone has the potential to overestimate aPCs.

To date, three methods for monitoring MRD have been developed, namely, MFC, allele specific quantitative PCR, and next-generation sequencing, and emerged as attractive and sensitive approaches^6,12,13^. Among these methods, EuroFlow-NGF is currently the most cost-effective and widely available method. Compared with the EuroFlow 2-tube method (< 350 USD/sample), the DuraClone single-tube method (< 100 USD/sample) is expected to be more cost-effective and can be used under public medical insurance in countries such as Japan.

In summary, we found Duraclone is a good option to evaluate MRD as the EuroFlow 8-color 2-tube method in MM but it should be used with caution and some discrepancies between them may have resulted because of the difference in the number of cells analyzed and antibody panels. Therefore, a sufficient cell acquisition number (> 5 × 10^6^) is essential to achieve high sensitivity in MRD detection in MM.

## Data Availability

The datasets generated during and/or analyzed during the current study are available from the corresponding author on reasonable request.
